# A community-based study on prevalence and correlates of erectile dysfunction among Kinondoni District Residents, Dar Es Salaam, Tanzania

**DOI:** 10.1186/s12978-016-0249-2

**Published:** 2016-11-29

**Authors:** Pedro Pallangyo, Paulina Nicholaus, Peter Kisenge, Henry Mayala, Noel Swai, Mohamed Janabi

**Affiliations:** 1 Department of Adult Cardiovascular Medicine, Jakaya Kikwete Cardiac Institute, P.O. Box 65141, Dar es Salaam, Tanzania; 2 Unit of Research, Jakaya Kikwete Cardiac Institute, P.O. Box 65141, Dar es Salaam, Tanzania

**Keywords:** Erectile dysfunction, Diabetes, Hypertension, Excess body weight, Community-based

## Abstract

**Background:**

Globally, erectile dysfunction burden (ED) is rising appreciably and it is projected to affect about 332 million men by the year 2025. This rise is attributable to the rising incidence of conditions associated with ED including obesity, diabetes, hypertension, coronary artery disease and depression. We conducted this community-based screening to elucidate on the prevalence of ED and its associated factors among men residing in an urban community in Tanzania.

**Methods:**

We conducted a cross-sectional community-based study and interviewed 441 men aged at least 18 years. Diabetes and hypertension were defined as per the International Diabetes Federation (IDF) and the 7th Report of the Joint National Committee (JNC 7) respectively. The 5-item version of the International Index of Erectile Function (IIEF-5) Scale was used to assess for erectile dysfunction. Multivariate logistic regression analyses were performed to explore the factors associated with ED.

**Results:**

The mean age was 47.1 years, 57.6 % had excess body weight, 8.2 % had diabetes and 61.5 % had high blood pressure. Overall, 24 % (106/441) of men in this study had some form of ED. Participants with age ≥55, positive smoking history, obesity, diabetes and hypertension displayed highest rates of ED in their respective subgroups. However, age ≥40 and diabetes were ultimately the strongest factors for ED after multivariate logistic regression analyses, (OR 5.0, 95 % CI 2.2–11.2, *p* < 0.001 and OR 5.3, 95 % CI 2.2–12.7, *p* < 0.001 respectively).

**Conclusion:**

Erectile dysfunction affects about a quarter of adult men living in Kinondoni district. Old age, obesity, smoking, hypertension and diabetes have the potential to increase the odds of ED up-to 5 times. In view of this, men with diabetes and hypertension should be offered screening services and treatment of ED as an integral component in their management.

## Plain English summary

Erectile dysfunction (ED) is defined as persistent inability to attain and/or maintain an erection sufficient for satisfactory sexual activity. ED is associated with old age and comorbidities including diabetes and hypertension amongst others. The incidence of ED is increasing globally and this is attributed to the aging population and the increase in the incidence of diabetes and hypertension. We aimed to determine the burden of ED among male residents of Kinondoni district, Dar es Salaam Tanzania. We recruited and interviewed 441 men aged at least 18 years. We utilized the 5-item version of the International Index of Erectile Function (IIEF-5) Scale for erectile dysfunction assessment. We defined diabetes and hypertension according to the International Diabetes Federation and the 7th Report of the Joint National Committee (JNC 7) respectively. About a quarter of all men had some form of ED. Old age and diabetes were associated with a 5 times increased likelihood for having ED. We concluded that ED is a common problem among men of reproductive age and that men particularly those with diabetes and hypertension should be offered screening services and treatment of ED as an integral component in their management.

## Background

Erectile dysfunction (ED) is defined as persistent inability to attain and/or maintain an erection sufficient for satisfactory sexual activity [1]. Despite its benign nature, ED has the potential to impair personal interactions, quality of life and well-being of both patients and their partners [2–4]. The rates of ED increase with age almost always indicating endothelial dysfunction [5–14]. Furthermore, ED is associated with a number of medical conditions or their treatment including diabetes mellitus, hypertension, coronary artery disease and depression [15–22].

Establishing the burden of ED in the community is challenging and most clinicians lack adequate skills in detecting and/or managing it [23, 24]. The reported rates worldwide have a wide variability ranging between 2 % and 90 % depending on the age, race, comorbidities, hospital- versus community-based, assessment tool, and geographical location of the population studied [6–8, 12, 25–38]. With the rising of comorbidities associated with ED worldwide, it is projected that its incidence will rise appreciably and that by year 2025, 332 million men will have some form of ED [39]. Similar to other developing nations, Tanzania is also witnessing an upsurge of non-communicable diseases with ED as an inevitable complication. Little is known about the community burden of ED in Tanzania. To elucidate on the prevalence of ED and its associated factors in a Tanzanian community we undertook this cross-sectional study among Kinondoni district men.

## Methods

### Study procedures & definition of terms

We conducted this 4-days community-based cross-sectional screening among men residing in Kinondoni district, Dar es Salaam, Tanzania in January 2016. Four hundred forty-one men of African descent aged at least 18 years were recruited and screened for erectile dysfunction. Participants were consented to participate in the study after they voluntarily came to the screening grounds for an organized general health check-up. Interviewers, mainly clinicians and nurses from the Jakaya Kikwete Cardiac Institute (JKCI) and Mwananyamala District Hospital were recruited and trained to administer the questionnaire and perform anthropometric, blood pressure (BP) and random/fasting blood glucose (RBG/FBG) measurements. Weight and height were measured with standard scales and BMI was calculated by a ratio of weight (in kilograms) to height (in meters) squared. WHO BMI cut-off values were used to define underweight, normal, overweight and obese [40]. Smoking history was sought and participants were categorized as current, past or a never smoker. Diabetes was diagnosed using RBG ≥11.1 mmol/L and/or FBG ≥7 mmol/L [41]. Prediabetes was defined as FBG of 5.6–6.9 mmol/L and/or RBG of 7.8–11.0 mmol/L [41]. Blood pressure was measured by digital BP machines where a systolic blood pressure (SBP) <120 mmHg and a diastolic blood pressure (DBP) <80 mmHg defined normotension. Pre-hypertension was defined by SBP of 120–139 mmHg or DBP of 80–89 mmHg, while SBP ≥140 mmHg or DBP ≥90 mmHg indicated hypertension [42]. The validated Swahili-translated 5-item version of the International Index of Erectile Function (IIEF-5) Scale was used to assess for erectile dysfunction [43–46]. The IIEF-5 Scale categorizes ED into five categories depending on the score i.e. 22–25: no ED, 17–21: mild ED, 12–16: mild to moderate ED, 8–11: moderate ED, 5–7: severe ED.

### Sample size calculations and recruitment process

This study was conducted in Kinondoni district which has an adult (≥18 years) population of about 1.633 million, 48.5 % of whom are males. Overall, we aimed to screen (for hypertension and diabetes) a number representing at least 0.1 % of the district’s total population i.e. ≥1633 persons. As this was the first local community-based study intending to investigate on the burden of ED, the authors hypothesized the prevalence to be 50 % and so it was predetermined that a minimum of 396 men (i.e. 50 % of the estimated 792/1633 men to be interviewed) will be assessed for ED. However, we exceeded the target and screened 1826 persons of whom 882 (48.3 %) were males. Our new minimum target for ED screening was then raised to 441 (i.e. 50 % of the recruited 882/1826 men), which is the exact number that comprised this study.

### Statistical analysis

Continuous variables are summarized and presented as means (± SD) while categorical variables are displayed as frequencies (percentages). Chi square tests and Student’s *T*-test were used in comparison of categorical and continuous variables respectively. Bivariate analyses were performed to determine factors associated with ED. Significant factors on bivariate analysis were included in a logistic regression model to control for confounders. Odd ratios with 95 % confidence intervals and *p*-values are reported. STATA v.11.0 was used for analysis, significance was set at *p* < 0.05 and all analyses were two-sided.

## Results

Socio-demographic and clinical characteristics of 441 study participants is displayed in Table 1. The mean age was 47.1 (±15.4) years and 63.7 % were aged 40 years and above. 53.8 % had completed primary school, 71 % were married, and 14 % ever smoked. The mean BMI was 26.6 (±5.3) kg/m^2^ and 57.6 % of individuals had excess body weight (i.e. BMI ≥25). The mean blood glucose level was 6.1 (±2.2) mmol/L and 8.2 % had type-2 diabetes mellitus. The mean SBP and DBP were 146 (±32) mmHg and 91 (±20) mmHg respectively and 61.5 % had hypertension.

**Table 1:** Socio-Demographic & Clinical Characteristics of Study Participants (*N* = 441)

Characteristic	n (%)
Age groups	
18–39	160 (36.3 %)
40–54	135 (30.6 %)
≥ 55	146 (33.1 %)
Education level	
None	12 (02.7 %)
Primary	237 (53.8 %)
Secondary	139 (31.5 %)
University	53 (12.0 %)
Marital status	
Single	95 (21.6 %)
Married	313 (71.0 %)
Divorced	24 (05.4 %)
Widowed	9 (02.0 %)
Smoking status	
Non-smoker	379 (86.0 %)
Current smoker	9 (02.0 %)
Past smoker	53 (12.0 %)
BMI status	
Underweight	14 (03.2 %)
Normal	173 (39.2 %)
Overweight	154 (35.0 %)
Obese	100 (22.6 %)
Blood Sugar Range	
Normal	344 (77.9 %)
Prediabetes	61 (13.9 %)
Diabetes	36 (08.2 %)
Blood Pressure Range	
Normal	60 (13.6 %)
Prehypertension	110 (24.9 %)
Hypertension	271 (61.5 %)

**Fig. 1:**
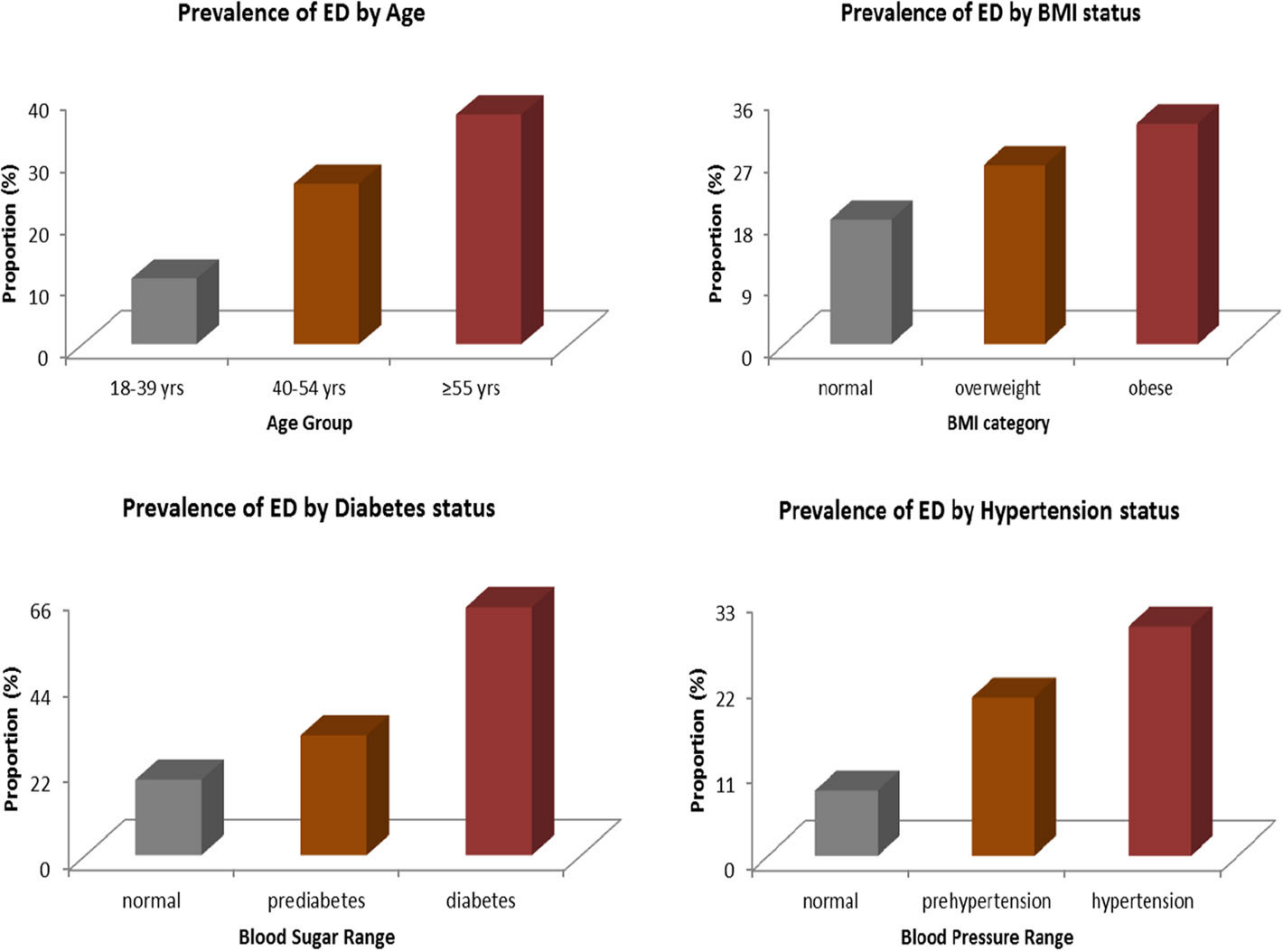
Prevalence of ED by Age, BMI, Diabetes and Hypertension status

Overall, 24 % (106/441) of men in this study had some form of ED. Of these, 9(8.5 %) had the mild form, 24 (22.6 %) mild-moderate form, 40 (37.8 %) moderate form, and 33 (31.1 %) had the severe form. Prevalence of ED increased with increase in age and weight i.e. 37 % and 32 % of those aged ≥55 and obese respectively had ED compared to 10.6 % and 18.1 % among those aged 18–39 years and normal BMI, (*p* < 0.001 and *p* < 0.01 respectively), Fig. 1. Men with a positive smoking history had a 40 % increased likelihood for ED compared to never smokers, (OR 1.4, 95%CI 0.7–2.5, *p* > 0.05). 63 % of men with diabetes had ED compared to 30.4 % with prediabetes and 19.1 % with normal blood glucose, (*p* < 0.01 and *p* < 0.001 respectively). Participants with prehypertension (20.2 %) and hypertension (29.3 %) had significantly higher ED rates compared to normotensive persons (8.3 %), *p* < 0.05 and *p* < 0.001 respectively.

Six variables including age, BMI, physical activity, smoking, hypertension, and diabetes status underwent bivariate analyses to assess if they have associations with ED. Four variables including age, BMI, hypertension and diabetes status revealed significant associations and these were added in a logistic regression model for further analysis. Table 2 displays results of multivariate logistic regression analysis. Men aged 40 years and above displayed a 5 times increased likelihood of having ED compared to those younger than 40 years, (OR 5.0, 95 % CI 2.2–11.2, *p* < 0.001). Likewise, diabetes was associated with a 5 times increased odds of having ED, (OR 5.3, 95 % CI 2.2–12.7, *p* < 0.001).

**Table 2:** Multivariate Logistic Regression Analysis of Factors Associated with ED

Test group	Comparative group	OR	95 % CI	*P*-value
Age ≥40	Age < 40	5.0	2.2–11.2	<0.001
BMI ≥30	BMI <30	1.4	0.7–2.5	0.306
Hypertensive	Non-hypertensive	1.2	0.6–2.3	0.541
Diabetic	Non-diabetic	5.3	2.2–12.7	<0.001

## Discussion

Nearly a quarter of men in this present study had ED with over two-thirds exhibiting the moderate to severe form. Our findings are close to a Chinese study by Bai et al. which produced a prevalence of 28.3 % [47]. A wide variability in the prevalence of ED from 13.2 % [48] in Egypt to 51.3 [25] in Singapore observed among studies [12, 25, 40, 49] is largely a result of variabilities in population characteristics and tools used for ED assessment among studies. Old age has been consistently shown to be a strong predictor of ED [25]. In this study, the rate of ED among participants aged 55 and above was three times compared to those in the age group 18–39 years. These findings echo the results of a landmark Massachusetts Male Aging Study [25] among others [2, 18, 29]. Aging is associated with comorbidities resulting into atherosclerosis and ultimately vascular dysfunction with ED as one of the manifestations [50].

Obesity was significantly associated with ED on bivariate analyses in this study. Such findings are in unison with Moreira [12] et al. study in which obesity proved to be a significant predictor on bivariate analysis but lost its significance after multivariate analysis. A study by Chung et al [51] showed that obesity is not an underlying factor for ED per se, but it does increase the risk through development of chronic vascular disease. Numerous studies [12, 50, 52–55] have suggested a dose dependent relationship between smoking and ED. Ever smokers in this study had a 40 % increased likelihood for ED compared to never smokers. A systematic review by Cao [55] et al. found that ED was increased by 20 % and 51 % among past- and current-smokers respectively. Apart from its potential to cause direct toxic effects on the vascular endothelium, smoking is linked to ED through its potential to mediate systemic changes including hypercoagulability, platelet aggregation and thromboxane-prostacyclin imbalance [56].

Diabetes is a well-established factor for ED. Participants with diabetes in our study had a 5 times increased odds for ED (OR 5.3) compared to diabetes-free persons. These current results have replicated the findings of a study by Zedan [57] et al. among Egyptian men which found an odds of 5.4. Other studies have consistently produced higher ED rates among diabetics compared to diabetes-free persons ranging between 35 % and 75 % [8, 11, 12, 25, 39, 58–63]. Diabetes is a risk factor for ED through its potential for causing autonomic neuropathy, gonadal dysfunction, and vascular and neurogenic impairment of penile smooth muscle [59, 64, 65]. Hypertensive participants had a tripled likelihood of having ED compared to their normotensive counterparts (*p* < 0.001), however the significance was lost after multivariate logistic analyses. Hypertension and some antihypertensive drugs have been shown to increase the risk for ED [17, 29]. High blood pressure is known to interfere with blood flow to the corpora cavernosa by causing narrowing and loss of elasticity of arteries thus resulting to ED [66].

Other factors including heavy alcohol consumption [2, 52], depression [18, 67–69], and low economic status [18, 70, 71] have been associated with increased risk for ED but were beyond the scope of this study. Further studies on ED in this area could investigate on their association with ED among Tanzanian men.

This study has several strengths including; (i) the use of an internationally recognized tool for assessing ED that will make comparison with other studies feasible, (ii) the simultaneous assessment of obesity, diabetes and hypertension allowed us to confirm the presence of these risk factors rather than relying on participants’ self-report. This study has some limitations as well, including; (i) the generalizability of our findings may be limited because the men screened in this study voluntarily came for screening and this might reflect a selected population of those either with a very good health seeking behavior or those having some health concerns necessitating medical help, (ii) Our assessment of ED was through interviews and we are aware that conditions like ED are highly associated with social stigmas. As a result of this, it is likely that ED was underreported by study participants, (iii) we made diagnoses of hypertension and diabetes based on a single point measurement of BP and RBG/FBG, thus our diabetes and hypertension rates might be overestimated, and (iv) our study was prone to several forms of bias including selection bias and non-differential bias, inevitably due to its cross-sectional nature.

## Conclusions

Several conclusions can be drawn from this present study: (i) ED affects one in four men over 18 years in Kinondoni district, (ii) age and diabetes mellitus are the strongest factors associated with ED, (iii) the high rates of ED among hypertensive and diabetic patients suggest that patients with such comorbidities should be screened for ED, (iv) with the increasing incidence of obesity, hypertension, diabetes and an aging population, ED may become a significant public health problem. In view of these findings: (i) services for diagnosis and treatment of ED should be incorporated in diabetes and hypertension clinics particularly in the developing world, and (ii) health programs should be designed in developing nations to educate and empower individuals on healthy eating, physical activeness and health seeking behavior.
